# Importance of Early Spotting of Diabetic Retinopathy in Type 2 Diabetes Patients by Family Medicine Physicians and Ophthalmologists: A Study in Jordan

**DOI:** 10.7759/cureus.34342

**Published:** 2023-01-29

**Authors:** Latifa Marie, Mohammad Al-Dabbas, Ahmed Khatatbeh, Ali Al-Mahmood

**Affiliations:** 1 Department of Medicine, Al-Balqa Applied University, Al-Salt, JOR; 2 Department of Ophthalmology, Jordanian Royal Medical Services, Amman, JOR; 3 Department of Ophthalmology, King Hussein Medical Center, Amman, JOR; 4 Department of Ophthalmology, Eye Specialty Hospital, Amman, JOR

**Keywords:** awareness, ophthalmologist, type-ii diabetes mellitus, family physician (fp), diabetic retinopathy

## Abstract

Background: Diabetes mellitus is a long-standing progressive disorder. Diabetic retinopathy is the primary cause of blindness among adults suffering from diabetes. Diabetic retinopathy is found to be dependent on the length of the period affected by diabetes, glucose control, blood pressure, and lipid profile while age, sex, and type of medical therapy were not found to be risk factors.

Aim: This study attempts to determine the importance of early spotting of diabetic retinopathy in Jordanian type 2 diabetes mellitus (T2DM) subjects by family medicine and ophthalmologist physicians, which will help us achieve better health outcomes.

Methods: Our retrospective investigation recruited 950 working-age subjects, of both sexes and with T2DM at three hospitals in Jordan, from September 2019 to June 2022. Early spotting of diabetic retinopathy was done by family medicine physicians and confirmation was done by ophthalmologists using direct ophthalmoscopy. Evaluation of the fundus by pupillary dilation was performed to assess the degree of diabetic retinopathy, macular edema, and the number of patients with diabetic retinopathy. The level of severity for diabetic retinopathy at confirmation was done using the classification for diabetic retinopathy produced by the American Association of Ophthalmology (AAO). Continuous parameters and independent t-tests were used to assess the average discrepancy in the degree of retinopathy among subjects. Categorical parameters were mentioned in numbers and percentages and chi-square tests were done to determine discrepancies in proportion among patients.

Results: Early spotting of diabetic retinopathy was recorded by family medicine physicians in 150 (15.8%) of 950 patients with T2DM of whom 56.7% (85/150) were women with an average age of 44 years. Of these 150 subjects with T2DM, who were presumed to have diabetic retinopathy, ophthalmologists diagnosed diabetic retinopathy in 35 patients (35/150; 23.3%). Of these, 33 (94.3%) had non-proliferative diabetic retinopathy and two (5.7%) had proliferative diabetic retinopathy. Of the 33 patients with non-proliferative diabetic retinopathy, 10 had mild non-proliferative diabetic retinopathy, 17 had moderate non-proliferative diabetic retinopathy, and six had severe non-proliferative diabetic retinopathy. Subjects aged more than 28 years had a 2.5 times increased risk of experiencing diabetic retinopathy. Awareness and lack of awareness values differed significantly (316 (33.3%), 634 (66.7%); P<0.05, respectively).

Conclusions: Early spotting of diabetic retinopathy by family medicine physicians shortens the delay of diagnosis confirmation by ophthalmologists.

## Introduction

Diabetes mellitus is a long-standing degenerative disorder with a prevalence of 2-6% worldwide [1,2]. While there are several metabolic, vascular, and neurological hazards induced by diabetes [3], diabetic retinopathy (DR) is one of the prominent ones, which is also the primary factor of blindness in this population [4]. DR is vascular with features of retinal ischemia (micro-aneurysms, hemorrhages, exudates, intra-retinal microvascular anomalies, anomalies in the venous caliber, and neovascularization) and of high vascular permeability, escalating from mild non-proliferative disease to moderate or severe non-proliferative retinopathy and ultimately proliferative disease [4].

Blindness originates from retinal hemorrhages in the fragile new vessels from the superficial capillary plexus (flame or splinter) or from beneath (mottled or stained); vitreous hemorrhage, macular edema, or retinal capillary hypo-perfusion with scarring and insult to the secondary retina [4]. Stimulation of the local renin-angiotensin system in the eyes of diabetic patients may directly or indirectly lead to an increase in the growth factor concentration in the vascular endothelium with angiogenesis and vasopermeability [5]. Macular edema is induced by pathological permeability of microcirculation with hard exudates shaped by lipoproteins, soft exudates, and myocardial signals in the nerve fiber layer as microaneurysms and microhemorrhages. Macular edema happens with retinopathy and is the first factor of reduced vision [6].

DR depends on the period of diabetes, glucose control, blood pressure, and lipid profile, while age, medical therapy, and sex are not risk factors [7]. Patients with type 2 diabetes mellitus (T2DM) need a yearly evaluation by an ophthalmologist to confirm DR to ensure the administration of early therapy.

The aim of our investigation was to determine the importance of early spotting of DR in patients with recently confirmed T2DM patients by family medicine physicians and ophthalmologists in Jordan.

## Materials and methods

Our retrospective investigation included 950 working-age subjects of both sexes who were recently confirmed with T2DM at three hospitals in Jordan, King Hussein Medical City (Amman), Salt Governmental Hospital (Al-Salt), and Eye Specialty Hospital (Amman), from September 2019 to June 2022. The study was carried out after obtaining written informed consent from all participants and verbal approval from the Deanship of Scientific Research and Innovation, Al-Balqa Applied University, Al-Salt, Jordan. 

Early spotting of diabetic retinopathy was done by family medicine physicians and confirmed by ophthalmologists using direct ophthalmoscopy. Evaluation of the fundus by pupillary dilation was performed to assess the degree of DR and macular edema, and the number of subjects with DR. The level of severity for DR at confirmation was ascertained using the classification for DR of the American Association of Ophthalmology (AAO). Fundus photos were taken following dilatation of the pupil with mydriacyl. Five 45° areas with a stereo pair of the macula were done with a fundus camera preset internal fixation. Two 30° photos were done of the optic disc centered on the macula. An ophthalmologist graded the fundus photos. DR was graded with a modification of the early treatment DR study adaptation of the modified Airlie House classification of DR. Eyes were graded based on: 0 (No DR; levels 10-13); 1 (non-proliferative DR: Mild; levels 14-20); 2 (Moderate; levels 31-43); 3 (severe; levels 47-53), and 4 (Proliferative DR; levels 60-85). DR was graded as levels 14-85. Diabetic macular edema was labeled if there are hard exudates and/or retinal thickening within a one-disc diameter of the center of the macula. Macular edema was graded as either present or absent. If data from both eyes were taken, the grading was based on the more severely affected eye.

Subjects had their visual acuity recorded with a pinhole test. Corrected visual acuity was recorded using auto-refractors with the built-in Snellen charts from 20 ⁄200 to 20 ⁄20. Retinal photos were taken in both eyes following mydriasis with a digital retinal camera. The anterior segment was evaluated for cataract and rubeosis and an examination of the fundus by pharmacological mydriasis was performed using an ophthalmoscope and lens with three mirrors in subjects with thickening of the macula. DR level was assessed based on AAO classification. Clinical significant macular edema was confirmed if thickening within 500 μ of the center of the macula, with exudates within 500 μ from the center of the macula, correlated with thickening or thickening of a disk or greater area, a disc diameter or less from the center of the macula. DR was classified and the most severe retinopathy was calculated. Subjects with a normal retina during fundus examination were advised for yearly follow-up of the fundus and subjects with any score of retinopathy were prescribed proper therapy.

Statistics

Continuous parameters and independent t-tests were used to assess the average discrepancy among subjects. Numbers and percentages were used to evaluate categorical parameters and Chi-square test was done to determine discrepancies in proportion among patients.

## Results

Early spotting of diabetic retinopathy was recorded by family medicine physicians in 150 (15.8%) of the total 950 T2DM patients, of whom 56.7% (85/150) were women with an average age of 44 years (Table 1). Of the 150 diabetes patients suspected by family medicine physicians to have DR, ophthalmologists confirmed DR in 23.3% (35/150). Of the 35 patients diagnosed with DR, 33 had non-proliferative DR and two (5.7%) had proliferative DR. Of the 33 patients with non-proliferative DR, 10 had slight non-proliferative DR, 17 had moderate non-proliferative DR, six had severe or very severe non-proliferative DR. Of the two patients with proliferative DR, one was in the early stage and one was in high-risk stage. There was no patient with advanced-stage proliferative DR (Table 2).

**Table 1:** Demographic data

Parameter		No.(%)
Gender	Male	65 (43.3)
	Female	85 (56.7)
Age (years)	<28	17 (11.3)
	>28	133 (88.7)
Diabetic retinopathy	Present	35 (23.3)
	Absent	115 (76.7)

**Table 2:** Diabetic retinopathy classification

Retinopathy		Number (%)
Non-proliferative	Mild	10 (28.6)
	Moderate	17 (48.6)
	Severe	4 (11.4)
	Very severe	2 (5.7)
Proliferative	Early	1 (2.9)
	High risk	1 (2.9)
	Advanced	0

A higher risk for DR was recorded in subjects recently diagnosed with T2DM and aged more than 28 years. Subjects aged more than 28 years had 2.5 times increased risk of experiencing DR (OR= 2.6; 95%CI: 0.22-17.8) and women had 1.4 times increased risk of having DR than men (OR= 1.5; 95%CI 0.82-2.0). Awareness of DR and induced blindness among participants is shown in Table 3. Awareness (n=316; 33.3%) was significantly less than non-awareness (n=634; 66.7%), P < 0.05.

**Table 3:** Awareness of diabetic retinopathy and induced blindness among participants

		Aware (n, %)	Not aware (n, %)	P-value
Number		316 (33.3)	634 (66.7)	
Gender	M	130 (41.1)	300 (47.3)	<0.05
	F	186 (58.9)	334 (52.7)	
Age (years)	<23	3 (0.9)	6 (0.9)	>0.5
	23-40	3 (0.9)	48 (7.6)	
	41-55	140 (44.3)	320 (50.5)	
	56-65	170 (53.8)	260 (41.01)	
Diabetic retinopathy period (years)	<2	3 (0.9)	55 (8.7)	<0.6
	2-4	41 (12.97)	160 (25.2)	
	4-8	50 (15.8)	130 (20.5)	
	>8	222 (70.3)	289 (45.6)	

## Discussion

DR is a major cause of vision loss among T2DM patients. Non-proliferative and proliferative DR in patients with T2DM are observed within five years of the progression of diabetes [3,5]. An incidence of 14.5% for non-proliferative and 1.6% for proliferative DR was found in patients confirmed with T2DM [8]. In our investigation, an incidence of 21.9% of non-proliferative and 1.9% of proliferative DR was recorded in subjects with recent confirmation of T2DM. Periodic follow-up (yearly or bi-yearly during the first five years) is more efficient than non-periodic follow-up in spotting DR in the middle and advanced ages but not in younger subjects. The follow-up model has no remarkable influence on DR-associated management in the five years' time. Strict follow-up in the first five years following confirmation of diabetes is not required according to previous studies [9].

DR is found in 63% of diabetics and increases the risk of loss of vision by 25 times. Ninety percent of subjects with T2DM and 65% of subjects with T2DM have retinopathy, 10 years following the start of the disease [3,7]. At least 25% of subjects recently confirmed with T2DM have retinopathy at disease confirmation [5,7]. While 40-45% of subjects with diabetes experience DR, the main common cause of blindness among subjects with diabetes [4,7]. Eighty-six percent of subjects with T2DM and 33% of subjects with T2DM had DR [8].

Even in patients who were aware of DR, there were deficits in data and attitude toward diabetes that delayed the diagnosis of DR. Awareness programs of DR should focus on retinal screening to decrease visual disability induced by diabetes [10].

DR starts a minimum of seven years before T2DM is confirmed symptomatically [1]. In a recently confirmed diabetes, there could be DR. It is mandatory to recognize subjects with retinopathy after diabetes confirmation and before vision is disturbed, as DR could be present even without eye features [11]. Protocol for effective evaluation of subjects with T2DM improves the quality of life. Direct ophthalmoscopy has a sensitivity of 80% and a specificity of more than 90% and is the technique of choice for confirmation of DR [4,11]. Nomograms might precisely anticipate the risk of DR in T2DM patients [12].

Primary care by family medicine physicians enhances the management of health services. Direct ophthalmoscopy must be done immediately by the family physician when diabetes is confirmed. Ophthalmological assessment sometimes is done only in subjects with chronic diabetes, or with visual disturbance, focusing on symptoms than signs. Our investigation ascertains the requirement for routine examination of the fundus in all recently confirmed diabetes, limiting insult to retinopathy, if correlated with proper glycemic control. DR was found in 20% of diabetes-confirmed patients. Ten percent of patients with diabetes had vision-compromising DR [1].

DR was remarkably correlated with a longer duration of diabetes, poor control, and microalbuminuria. Good glucose control at the time of confirmation of diabetes along with efficient treatment of retinopathy is crucial [13].

All females aged more than 30 years with a primary confirmation of T2DM must have an ophthalmological assessment [7]. The age of first confirmation of T2DM is related to a higher risk of DR, more so in females, where the risk increases by 3.9 times. It is important to show the requirement for direct ophthalmology for all diabetic patients and the assessment by an ophthalmologist with follow-up. Most patients with T2DM had moderate awareness, requiring enhanced awareness of the hazard on the retina and management choices. Retinal follow-up must be encouraged to decrease the risk of visual hazards. DR follow-up must not be restricted to eye care centers. Enhanced awareness will assist patients and healthcare providers to attain proper outcomes in the prophylaxis of DR [14]. Roto et al. reported that the prevalence of DR in Jordan was 28.2% [15]. This is considered to be slightly high when compared to the results in our study (23.3%); however, it should be taken into consideration that their study was conducted at the ophthalmology clinic, unlike our study which was performed at the family medicine clinic. In addition, Al-Latayfeh et al. found that most patients with diabetes were aware of diabetes management but not DR [16].

One limitation of our investigation is that we should have included the prevalence of other risk factors among patients like hypertension, hyperlipidemia, anemia, and glycated hemoglobin (HbA1C).

## Conclusions

DR is a feature of retinal ischemia and of high vascular permeability, escalating from mild non-proliferative disease to moderate or severe non-proliferative retinopathy and, ultimately, proliferative disease. DR relies on the period of diabetes, glucose control, blood pressure, and blood lipids. Patients with T2DM need a yearly evaluation to confirm the onset or progression of DR for early therapy. This is what makes our study special as it highlights the importance of early DR detection by family physicians, which will result in better outcomes.
